# A comprehensive framework for automated segmentation of perivascular spaces in brain MRI with the nnU-Net

**DOI:** 10.1007/s00234-026-03993-y

**Published:** 2026-04-17

**Authors:** William Pham, Alexander Jarema, Donggyu Rim, Zhibin Chen, Mohamed Khlif, Vaughan Macefield, Luke Henderson, Amy Brodtmann

**Affiliations:** 1 Department of Neuroscience, Monash University, Melbourne, Australia; 2 Department of Radiology, Alfred Health, Melbourne, Australia; 3 School of Medicine, Western Sydney University, Sydney, Australia; 4 Department of Anatomy and Histology, The University of Sydney, Sydney, Australia

**Keywords:** Perivascular spaces, nnU-Net, Deep learning, MRI, Virchow-Robin spaces, Convolutional neural networks, 3T, 7T, Segmentation, U-Net

## Abstract

**Background:**

Enlargement of perivascular spaces (PVS) is common in cerebral small vessel disease, Alzheimer’s disease, and Parkinson’s disease, reflecting impaired clearance pathways. While MRI provides a means to quantify perivascular spaces, manual annotation remains time-consuming and labour-intensive. Thus, there is a need for accurate automated MRI-based PVS segmentation methods.

**Aim:**

To optimise the nnU-Net, a deep-learning framework, for PVS segmentation.

**Methods:**

30 T1-weighted (T1w) MRI images acquired on three different scanners were used. PVS in the white matter (WM) and basal ganglia (BG) were manually labelled via a sparse annotation strategy and used to optimise the nnU-Net for T1w PVS segmentation. The same pipeline was applied to T2-weighted (T2w) images. Additionally, we trained T1w+FLAIR and T2w+FLAIR models to simultaneously segment PVS and white matter hyperintensities (WMH). Performance was assessed with 5-fold cross validation using the Dice similarity coefficient (DSC).

**Results:**

A voxel-spacing agnostic model (mean DSC = 64.3 ± 3.3%) outperformed models that resampled images to a common resolution (DSC = 40.5–55%). Training on PVS segmentations derived from preprocessed T1w images substantially improved performance (DSC = 78.3 ± 1.7%). The T2w model performed best overall (DSC = 84.7 ± 1.3%), especially for WM-PVS (DSC = 90.4 ± 0.9%) compared to BG-PVS (DSC = 79.1 ± 2%). Multimodal models achieved DSCs of 75.6 ± 3.4% (T1w+FLAIR) and 77.5 ± 2.7% (T2w+FLAIR).

**Conclusions:**

Our deep learning models provide a robust framework for automated PVS quantification across MRI modalities.

**Supplementary information:**

The online version contains supplementary material available at 10.1007/s00234-026-03993-y.

## Introduction

Perivascular spaces (PVS) are fluid filled channels between the vascular lumen, where blood flows, and the interstitial space surrounding vessels [1]. During certain physiological states, such as sleep, fluid and solute transport through perivascular spaces increases significantly [2], resulting in the enhanced clearance of molecular waste from the nervous system (i.e., glymphatic clearance) [3]. Impaired glymphatic clearance, observed in sleep deprivation and various disease states, is thought to exacerbate the accumulation of toxic metabolites in the brain, compromising neuronal health and cognition [4–6]. Enlarged PVS, an indicator of glymphatic dysfunction, are linked to multiple neurological diseases including cerebral small vessel disease (CSVD), ischaemic stroke, Alzheimer’s disease, multiple sclerosis, and traumatic brain injury [7–9].

Structural MRI allows in-vivo visualisation, providing a means to monitor disease-related changes and progression. Manual segmentation remains the gold standard for voxel-wise PVS delineation but is prohibitively laborious for large-scale studies. Advances in biomedical image processing have led to automated approaches to PVS segmentation from MRI [10, 11]. One of the most widely used architectures for biomedical image segmentation is the U-Net, comprising a contracting encoder for feature extraction followed by an expanding decoder for label generation [12]. Currently, the no-new-UNet (nnU-Net) is considered the benchmark for biomedical image segmentation [13]. It builds on the original U-Net architecture, using rule-based selection of hyperparameters, data handling, and image preprocessing [13]. The latest variant, called the nnU-Net Residual Encoder (ResEnc), incorporates residual connections within encoder convolutional blocks and has consistently outperformed earlier versions [14].

To enhance PVS visibility, image preprocessing methods such as denoising and contrast enhancement are commonly used. However, these techniques have not previously been integrated into machine learning models for PVS segmentation. Here, we applied the nnU-Net ResEnc for automated PVS segmentation in T1-weighted (T1w) and T2-weighted (T2w) MRI using an efficient sparse annotation strategy. To boost model performance, we implemented preprocessing techniques, including denoising with non-local means filtering (NLMF) and contrast adjustment with adaptive histogram equalisation (AHE). Finally, we developed multimodal models incorporating FLuid Attenuated Inversion Recovery (FLAIR) MRI to enable concurrent segmentation of white matter hyperintensities (WMH) alongside PVS, and T1w models dedicated to PVS segmentation in midbrain and hippocampal regions, which have not been targeted in previous work.

## Methods

### Primary datasets

#### Participants

Our primary training dataset comprised MRI data acquired from healthy participants (*n* = 30), recruited as part of three separate studies using three different imaging protocols and three different MRI machines. Ten participants were included from each dataset. We refer to these datasets as Dataset A, Dataset B, and Dataset C. Dataset A was curated with ethics approval from the Human Research Ethics Committee (HREC) of the University of Sydney [15]. Dataset B was curated with ethics approval from the HRECs of the University of Western Sydney and the University of New South Wales [16]. Dataset C is curated from the Cognition and Neocortical Volume After Stroke (CANVAS) study approved by respective ethics committees from three Stroke units at the Austin Hospital, Box Hill Hospital, and Royal Melbourne Hospital [17]. All participants provided informed written consent in accordance with the Declaration of Helsinki. Participants in Dataset A were the youngest (5 females, mean ± SD age = 28.3 ± 11.5 years) compared to Dataset B (5 females, mean age = 55.5 ± 10.4 years) and Dataset C (3 females, mean age = 66.1 ± 8 years).

#### MRI acquisition

T1-weighted (T1w) MPRAGE sequence images were available from all three datasets. T1w sequences in Dataset A were acquired on a 7 T Siemens, Magnetom model MRI scanner with a single-channel transmit and 32-channel receive head coil (Nova Medical) (TR = 5000 ms, TE = 3.1 ms, FA = 4°, FOV = 240 mm × 167.25 mm, voxel size = 0.75 mm isotropic). Dataset B was acquired on a 3 T Philips, Achieva model MRI scanner with a 32-channel SENSE head coil (TR = 5600 ms, TE = 2.5 ms, FA = 8°, FOV = 250.56 mm × 174 mm, voxel size = 0.87 mm isotropic). Dataset C was acquired on 3 T Siemens, Trio Tim model MRI scanners with a 12-channel head coil (Siemens, Erlangen, Germany). In addition to T1w sequence (TR = 1900 ms, TE = 2.55 ms, TI = 900 ms, FA = 9°, FOV = 256 mm × 256 mm, voxel size = 1.0 mm isotropic), Dataset C included a T2-weighted (T2w) sequence (TR = 3390 ms, TE = 390 ms, FA = 120°, FOV = 256 mm × 256 mm, voxel size = 1.0 mm isotropic), and a FLAIR sequence (TR = 6000 ms, TE = 380 ms, TI = 2100 ms, FA = 120°, FOV = 512 mm × 512 mm, voxel size = 1.0 mm isotropic) used to differentiate white matter hyperintensities from perivascular spaces. Supplementary Table 1 provides the MRI acquisition parameters for the three datasets. All available images from the healthy participants were visually inspected. From each dataset, ten participants with high PVS burden were selected for manual segmentation. Poor quality images due to motion artefacts, ghosting, Gibbs ringing, or phase-encoding artefacts were avoided.

### Secondary dataset

**Fig. 1:**
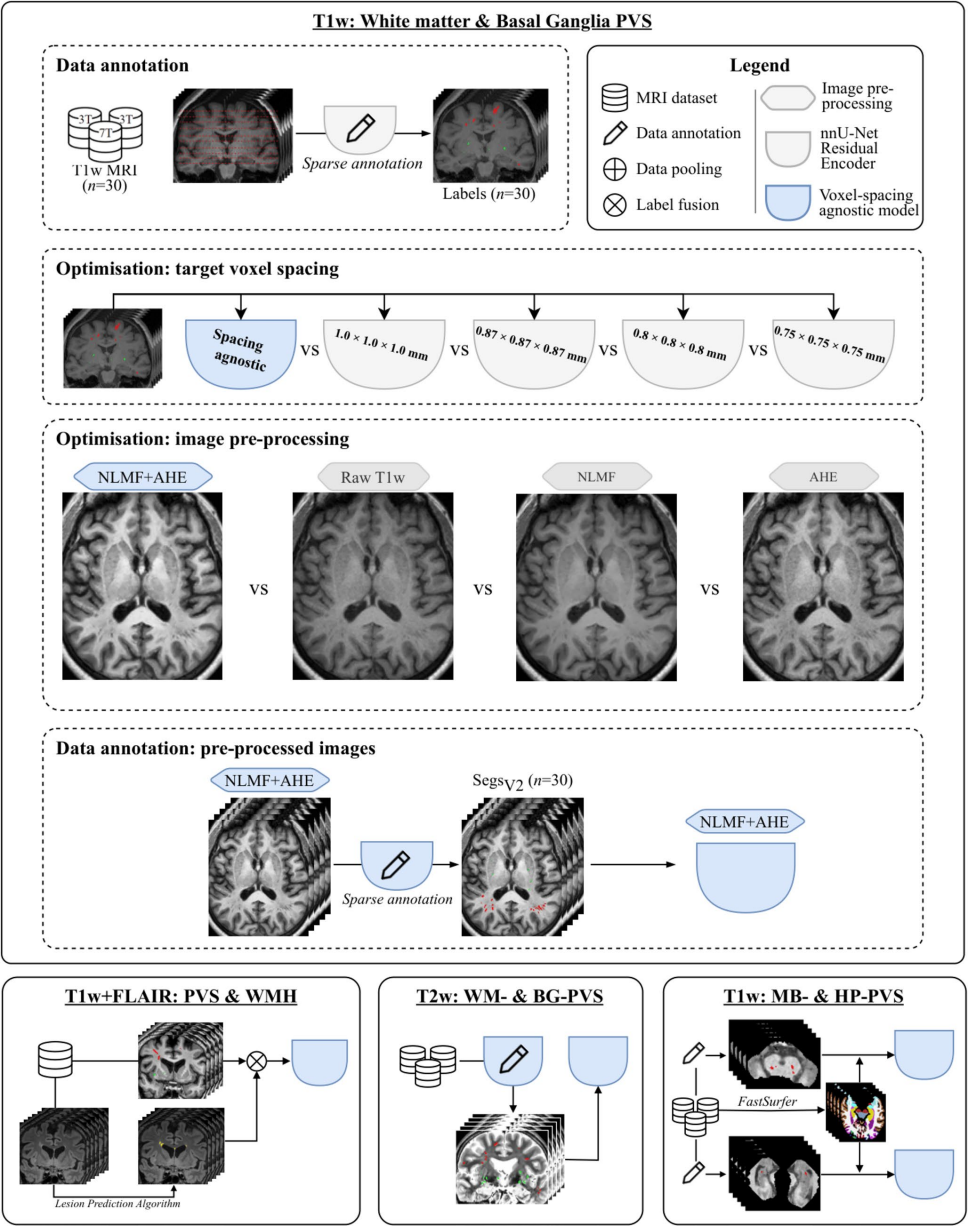
Overall workflow of the nnU-Net implementation. Panel 1: development of the nnU-Net PVS segmentation in the white matter (WM) and basal ganglia (BG) using T1-weighted (T1w) MRI. Sub-panels show the acquisition of initial labelled data with a sparse annotation strategy, followed by model optimisation by comparing image handling parameters, and preprocessing techniques. Panel 2: development of nnU-Net models using T1w and FLAIR images to segment WM and BG-PVS alongside white matter hyperintensities (WMHs). Panel 3: development of models using T2w images for the segmentation of WM and BG-PVS. Panel 4: development of nnU-Net models for PVS segmentation in the midbrain (MB) and hippocampi (HP). NLMF: non-local means filtering. AHE: adaptive histogram equalisation

Secondary datasets were incorporated to (1) supplement training dataset in the T2w and T2w+FLAIR nnU-Net models (Sect. 2.6–2.7), and (2) supplement training data for midbrain (MB) or hippocampal (HP) PVS segmentation to facilitate model convergence (Sect. 2.8). All MRI scans in the secondary dataset were used without preprocessing unless otherwise stated. The overall study workflow is shown in Fig. 1 and a summary of all trained models and their corresponding datasets is provided in Table 1.

**Table 1:** Summary of models and associated datasets used for training and validation. Each model was trained and evaluated in a 5-fold cross validation. The datasets consisted of images from healthy participants, except for five frontotemporal dementia cases from the NIFD dataset, which were included in training of the T2w and T2w+FLAIR models. WM: white matter. BG: basal ganglia. MB: midbrain. HP: hippocampus. WMH: white matter hyperintensities.

Modality	Targets	Datasets	Images (*n*)	Resolution
*T1w*	WM-PVS BG-PVS	A	10	7T; 0.75 mm
		B	10	3T; 0.87 mm
		C	10	3T; 1 mm
		**Total**	30	
*T1w+FLAIR*	WM-PVS BG-PVS WMH	C	10	3T; 1 mm
		**Total**	10	
*T2w*	WM-PVS BG-PVS	C	5	3T; 1 mm
		HCP1200	5	3T; 0.7 mm
		NIFD	5	3T; 1 mm
		**Total**	15	
*T2w+FLAIR*	WM-PVS BG-PVS WMH	C	5	3T; 1 mm
		NIFD	5	3T; 1 mm
		**Total**	10	
*T1w*	MB-PVS	A	10	7T; 0.75 mm
		B	10	3T; 0.87 mm
		C	10	3T; 1 mm
		ADNI3	10	3T; 1 mm
		AIBL	10	3T; 1 mm (*n*=8) 3T; 1.2 mm (*n*=2)
		OASIS-3	10	3T; 1 mm (*n*=6) 3T; 1.2 mm (*n*=4)
		**Total**	60	
*T1w*	HP-PVS	B	10	3T; 0.87 mm
		C	10	3T; 1 mm
		ADNI3	10	3T; 1 mm
		AIBL	10	3T; 1 mm (*n*=5) 3T; 1.2 mm (*n*=5)
		OASIS-3	10	3T; 1 mm
		**Total**	50	

Images from datasets C (*n* = 5), HCP1200 (*n* = 5), and the Neuroimaging Initiative for Frontotemporal lobar Degeneration (NIFD, *n* = 5) were used to train the T2w model (Sect. 2.6) [18]. As raw MRI scans from HCP1200 were unavailable, we used preprocessed images from the standard HCP pipeline, which included bias field correction and skull stripping [19]. The T2w+FLAIR model was trained using images from datasets C (*n* = 5) and NIFD (*n* = 5) (Sect. 2.7). Images from dataset C and NIFD were incorporated into our pipeline in raw and unprocessed form. Demographics and MRI parameters for all datasets used in model training are provided in Supplementary Tables 2–5.

Images from the ADNI3 (*n* = 10), AIBL (*n* = 10), and OASIS-3 (*n* = 10) datasets [20–22] were used to train nnU-Net models for MB- and HP-PVS segmentation (Sect. 2.8), with dataset characteristics reported in Supplementary Tables 6–9. A description of the secondary datasets is provided in Supplementary Methods Sect. 1.

### Model architecture

All models were trained using the 3D full-resolution nnU-Net with the Residual Encoder (“ResEncM”) configuration [23]. This differs from the original nnU-Net architecture without residual connections in the encoder pathway [13]. The Residual Encoder is a recent extension to the nnU-Net pipeline and has demonstrated improved performance over the original model. For brevity, we refer to the extended variant as “nnU-Net” throughout, unless otherwise specified.

By default, nnU-Net sets the target voxel-spacing to the median of the training data. Additionally, we tested customised voxel-spacings as well as a voxel-spacing agnostic configuration (Sect. 2.4.2). The latter assumes a uniform voxel resolution across all scans and therefore avoids resampling during preprocessing and inference. Standard nnU-Net preprocessing includes voxel intensity clipping to the 0.5th − 99.5th percentiles followed by z-score normalisation. All models used the default preprocessing, except those employing adaptive histogram equalisation (Sect. 2.4.3), which requires voxel intensities to be scaled to the range of (0, 1). Training data were augmented with the *batchgenerators* package [24]. Spatial augmentations included random mirroring, elastic deformations, rotations, and rescaling. Intensity augmentations included the addition of Gaussian noise.

### T1w nnU-Net: WM and BG-PVS

#### Sparse annotation strategy

**Fig. 2:**
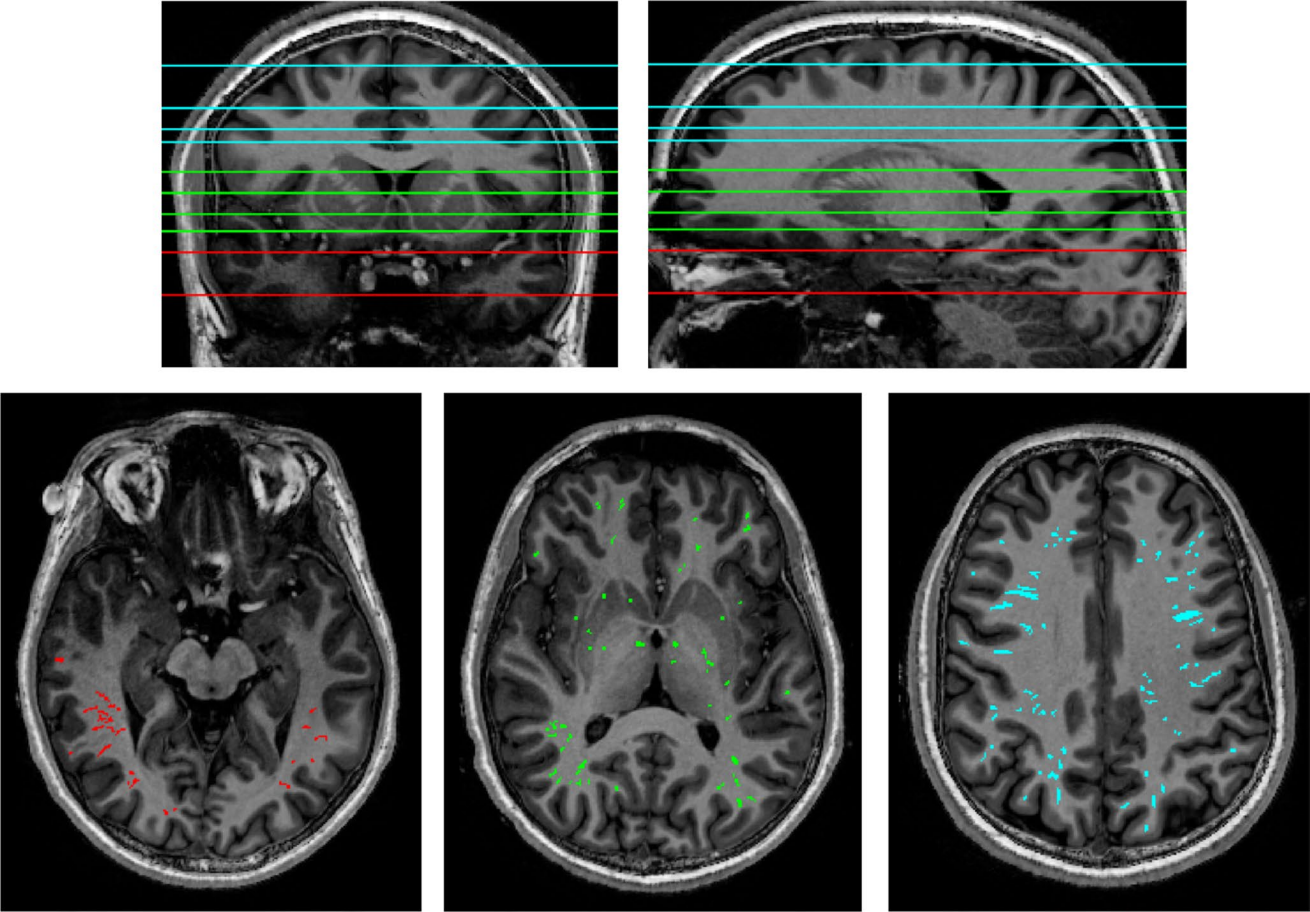
T1-weighted MRI demonstrating the sparse segmentation strategy. Ten Axial slices from each image were selected for manual segmentation (Top-Left: coronal slice; Top-Right: sagittal slice). Red bands indicate the two axial slices at the brainstem (BS) level. Green bands indicate the four axial slices selected at the basal ganglia (BG) level. Cyan bands indicate the four axial slices selected at the centrum semiovale (CS) level. Bottom: axial slices with MRI-PVS labelled

Ten T1w MRI scans from healthy controls were selected from each of the three primary datasets (A, B, and C). For each scan, PVS were manually labelled on ten axial slices by a single rater (WP), reviewed by an experienced radiologist (AJ), and revised if necessary (Fig. 2). An nnU-Net model was then trained using a sparse annotation strategy, where unlabelled axial slices were assigned an “ignore” label and the model was optimised with a partial loss function to generate complete segmentations [25].

The resulting segmentations were reviewed and manually corrected to ensure accuracy. PVS voxels were subsequently assigned region-specific labels: white matter (WM label = 1) and basal ganglia (BG, label = 2). All manual annotations were performed using ITK-Snap [26]. The set of 30 segmentations was then used to train and optimise a single nnU-Net pipeline for WM- and BG-PVS segmentation in T1w MRI.

#### Target spacing optimisation

We trained and evaluated nnU-Net models on all 30 labelled images to identify the optimal image preprocessing configuration. Four models using different voxel-spacing (0.75, 0.80, 0.87, and 1.00 mm isotropic), in addition to a voxel-spacing agnostic model, which ignores voxel-spacing information and avoids resampling of images, were compared. The best-performing voxel-spacing was selected for all subsequent models. All configurations were validated using stratified 5-fold cross-validation (5FCV).

#### Image preprocessing optimisation

**Fig. 3:**
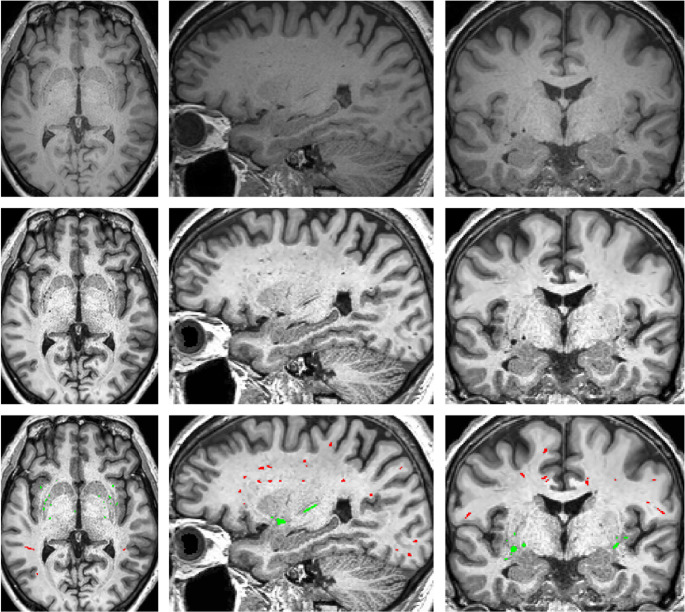
The effect of image enhancement on a T1w scan from Dataset B (3T, Philips Achieva, 0.87 mm isotropic voxel size). Top: raw T1w image. Middle: T1w image after non-local means filtering (NLMF) and adaptive histogram equalisation (AHE). Bottom: manual PVS segmentation overlaid on the preprocessed MRI scan with WM-PVS (red) and BG-PVS (green)

Next, we evaluated the effects of image preprocessing on PVS segmentation performance. The preprocessing pipeline consisted of voxel intensity rescaling to a range of 0–1, following by denoising with NLMF [27, 28], and/or contrast adjustment with AHE. All preprocessing was performed using Python (v3.9). NLMF was implemented using the *dipy* package [29], and AHE via the *scikit-image* package [30]. We trained the voxel-spacing agnostic nnU-Net under three conditions: (i) NLMF-only images, (ii) AHE-only images, and (iii) combined NLMF + AHE images (Fig. 3). Segmentation performance was evaluated for each condition using stratified 5FCV. The combined NLMF + AHE preprocessing enhanced PVS visibility. Therefore, these images were used to generate PVS segmentations via a sparse annotation strategy. Segmentations were manually corrected by a single rater (WP) and reviewed by an experienced radiologist (AJ). A new nnU-Net model was subsequently trained on the segmentations generated from the preprocessed images, and its performance was assessed via a stratified 5FCV conducted with the updated segmentations.

### T1w+FLAIR nnU-Net

As PVS may co-localise with white matter hypo-intensities on T1w MRI, we incorporated FLAIR images to help distinguish PVS from these lesions in the T1w+FLAIR model. We trained a voxel-spacing agnostic nnU-Net with T1w MRI and FLAIR scans registered to corresponding T1w images using the *ANTSRegistrationSyn* algorithm with rigid and affine transformations and nearest-neighbour interpolation [31, 32]. The model was trained on 10 images from dataset C. PVS segmentations derived from preprocessed T1w scans (Sect. 2.4.3) were combined with WMH masks generated by the Lesion Prediction Algorithm (LPA) in the Lesion Segmentation Tool [33]. This resulted in combined segmentations with distinct voxel labels: WM-PVS (label = 1), BG-PVS (label = 2), and WMH (label = 3). The T1w+FLAIR model was trained and evaluated in a 5FCV. WMH masks were generated with the LPA in SPM12 (MATLAB r2018a), binarised at a 0.5 threshold using *fslmaths* from FSL (v6.0.4) [34], and applied to identify and exclude false-positive PVS labels overlapping WMH regions.

### T2w nnU-Net

We trained and evaluated an nnU-Net for WM- and BG-PVS segmentation using 15 T2w images from datasets C, HCP1200, and NIFD. PVS segmentations were generated with a sparse annotation strategy, in which five axial slices per scan were manually labelled. Model generated labels were then refined by a single rater (WP) and reviewed by a radiologist (AJ). All T2w scans underwent preprocessing with NLMF and AHE. The model trained on these preprocessed scans was evaluated using a stratified 5FCV, with each fold comprising 12 training images (four per dataset) and three test images (one per dataset).

### T2w+FLAIR nnU-Net

**Fig. 4:**
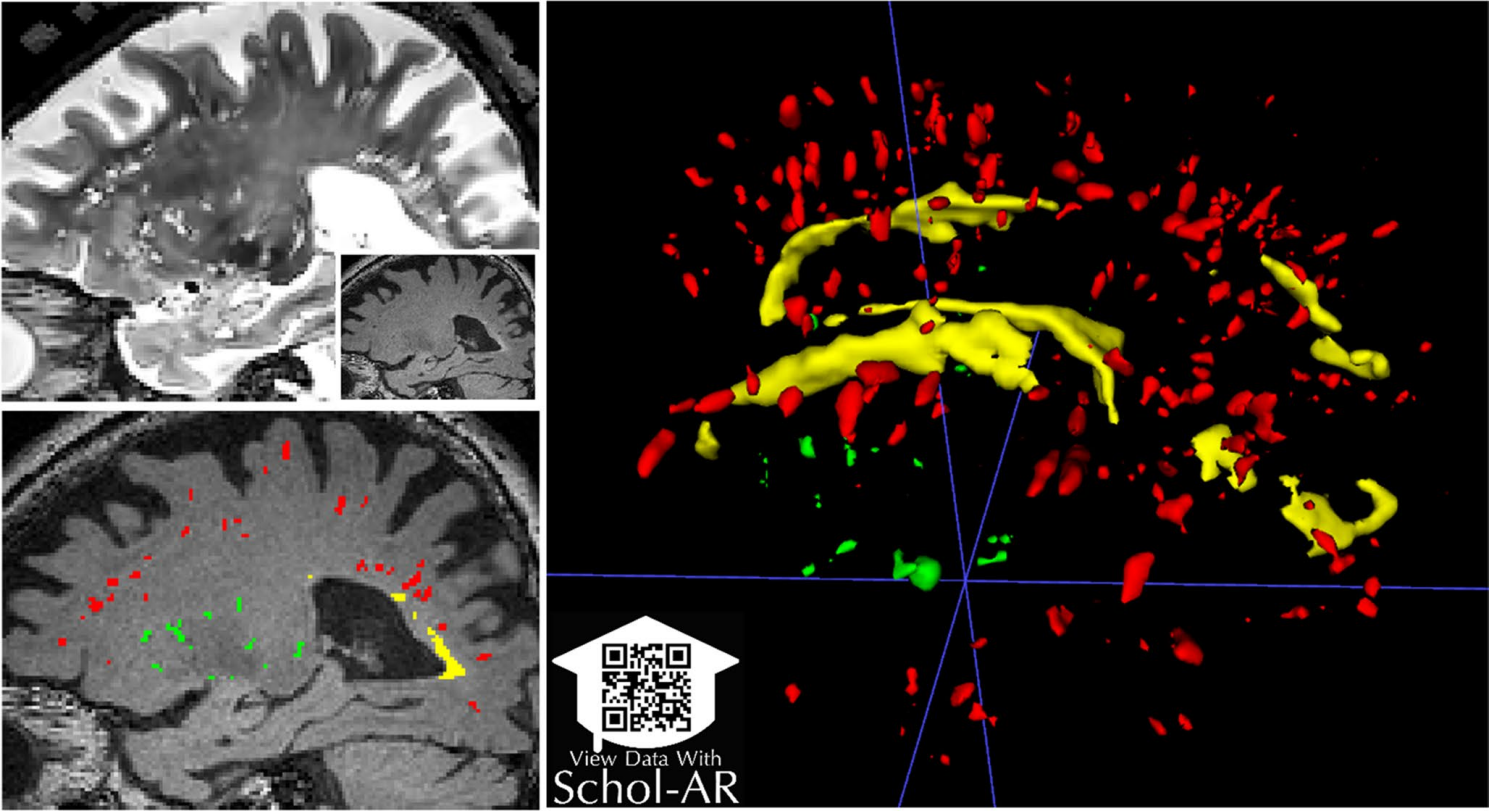
PVS segmentation from the NIFD dataset used to test the T2w+FLAIR model. Top-left: Raw T2w scan with corresponding FLAIR scan (inset). Bottom-left: PVS segmentation generated by the T2w+FLAIR nnU-Net overlaid onto the corresponding FLAIR scan with WM-PVS (red), BG-PVS (green), and white matter hyperintensities (yellow). Right: 3D render of the PVS segmentation augmented with Schol-AR and viewable via the QR code [35]

Given that PVS are hyperintense on T2w yet largely suppressed on FLAIR, whereas WMHs remain hyperintense on both T2w and FLAIR, incorporating FLAIR alongside T2w provides additional information to help the model distinguish PVS from WMH. We trained a multimodal voxel-spacing agnostic nnU-Net with T2w MRI and FLAIR scans similarly registered to T2w as described earlier. The model was trained on 10 images from datasets C and NIFD (5 images per dataset). PVS segmentations derived from preprocessed T2w scans (Sect. 2.6) were combined with WMH masks generated by LPA in the Lesion Segmentation Tool [33] to produce distinct voxel labels: WM-PVS (label = 1), BG-PVS (label = 2), and WMH (label = 3) (Fig. 4). The T2w+FLAIR model was trained and evaluated using a stratified 5FCV. The WMH maps were again used to identify and exclude false-positive PVS labels.

### T1w nnU-Net: MB and HP-PVS

**Fig. 5:**
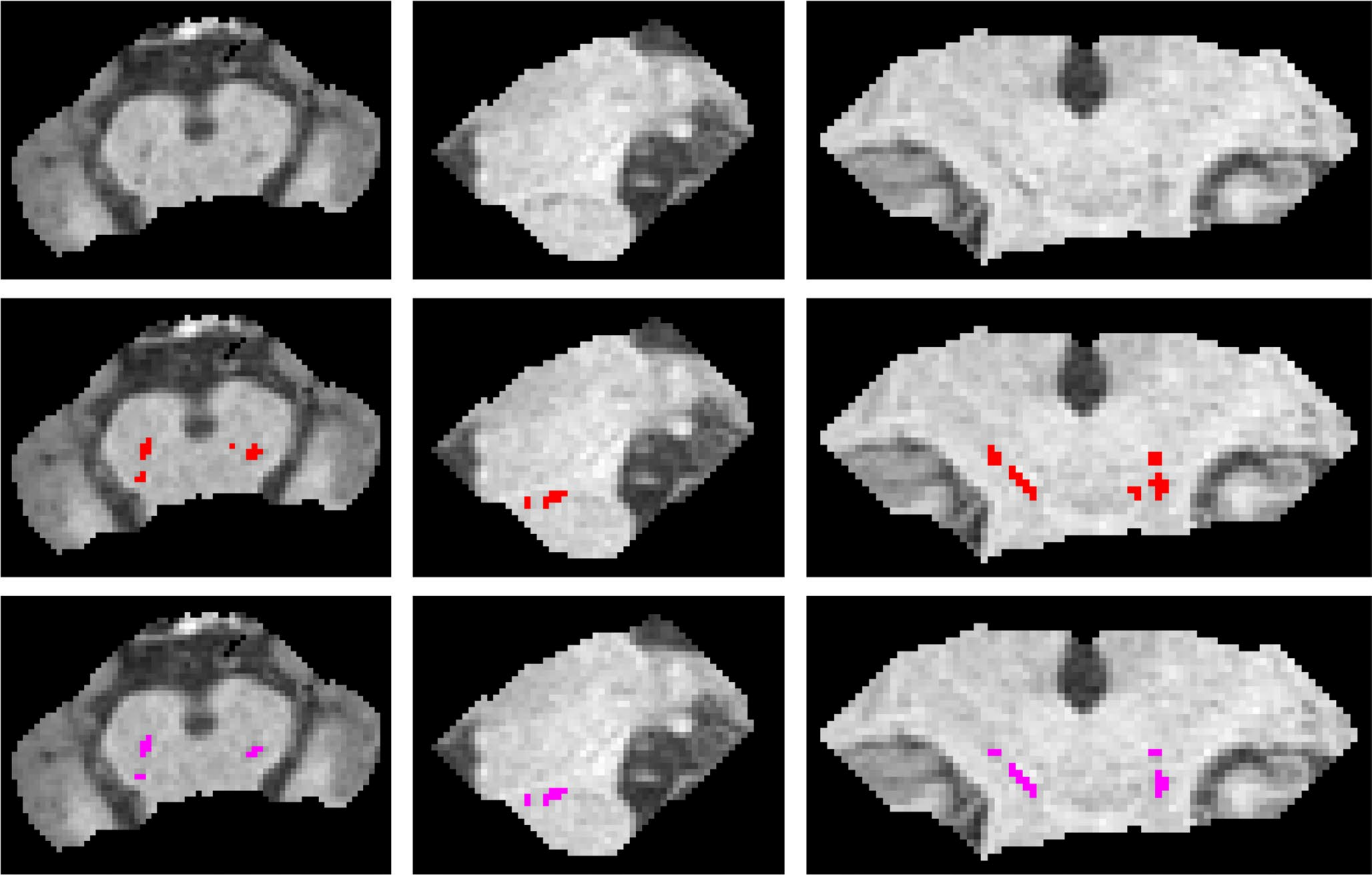
Axial slices from a T1w scan showing PVS labelled in the midbrain. Top row: raw images. Middle row: manually labelled PVS in red. Bottom row: automatically labelled PVS in magenta. Left column: axial plane. Middle column: sagittal plane. Right column: coronal plane

**Fig. 6:**
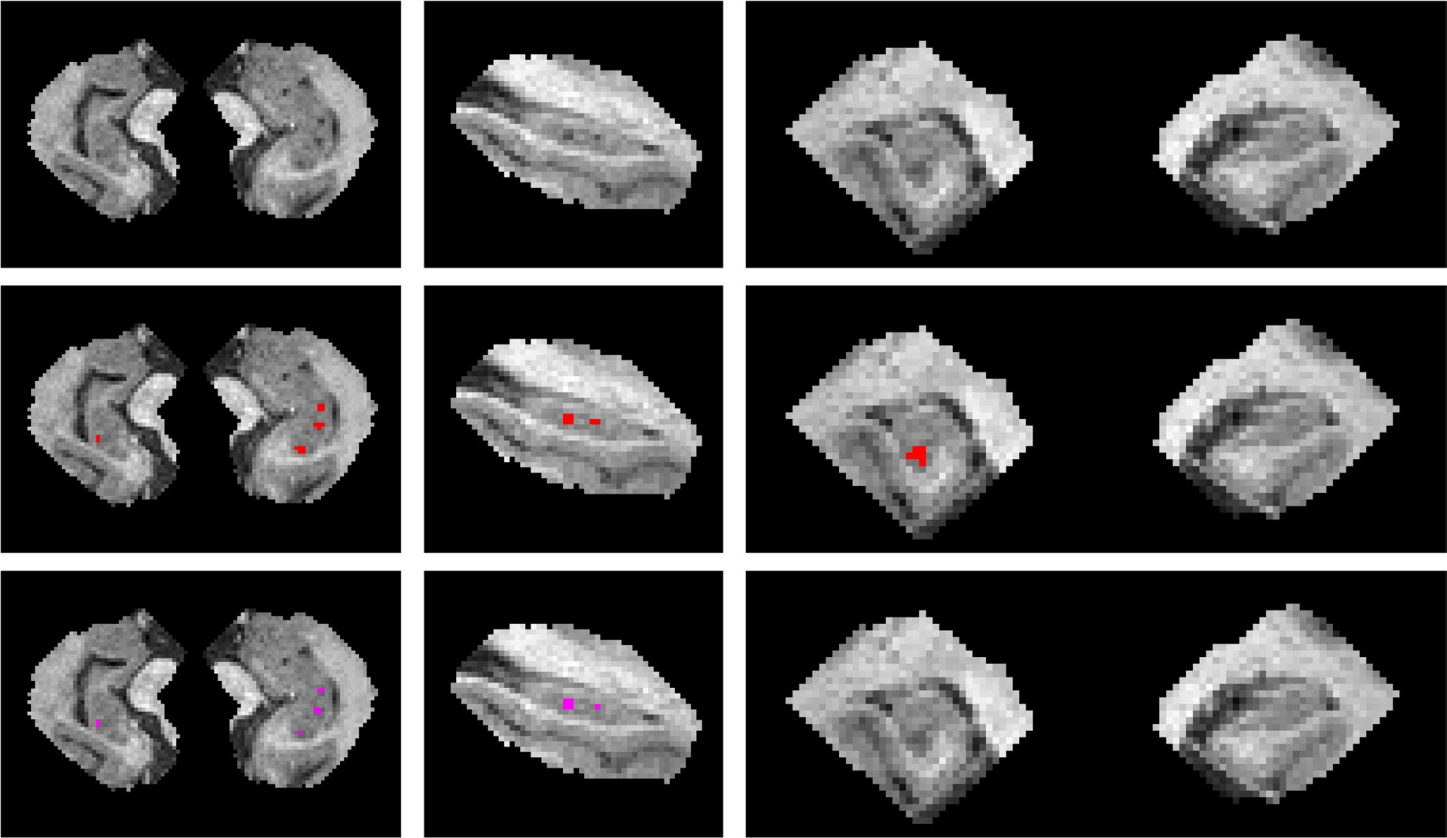
Axial slices from a T1w scan showing PVS labelled in the hippocampi. Top row: raw images. Middle row: manually labelled PVS in red. Bottom row: automatically labelled PVS in magenta. Left column: axial plane. Middle column: sagittal plane. Right column: coronal plane

We optimised two nnU-Net models for the segmentation of PVS in the midbrain (MB) and hippocampus (HP) using T1w MRI. The MB-PVS model was trained on images from 60 participants (28 females, mean age = 59.7 ± 17.3 years): 30 from the primary datasets, 10 from ADNI3, 10 from AIBL, and 10 from OASIS-3. The HP-PVS model was trained using 50 images (20 females, mean age = 68.2 ± 10.1 years): 10 from each of the datasets B, C, ADNI3, AIBL, and OASIS-3. Dataset A was excluded because no hippocampal PVS were visible. All MB- and HP-PVS were manually annotated by a single rater (WP) and reviewed by an experienced radiologist (AJ), following established guidelines (Figs. 5 and 6) [36–38].

An initial attempt to train a unified model for PVS segmentation in the white matter, basal ganglia, MB, and HP failed to converge, likely due to severe class imbalance from the relatively small MB and HP regions. We therefore tested three model variations: (i) whole T1w MRI, (ii) combining whole T1w MRI with FastSurfer (v2) parcellation masks [39], and (iii) partial T1w MRI restricted to regions of interest (ROIs). Only models from the third variation consistently converged across 1000 training epochs in each fold, and results are reported for this configuration.

For the final approach, FastSurfer parcellations registered to the Desikan-Killiany-Tourville atlas [39, 40] were combined with the whole T1w scans to isolate the MB and HP. For the MB-PVS model, the “ventral-diencephalon” ROI was retained. For the HP-PVS model, the “left hippocampus” and “right hippocampus” ROIs were retained. Binary ROI masks were dilated using the *binary_dilation* function in *scipy.morphology* (v.1.6.2) to ensure complete coverage of the structures.

### Model training

Models were trained with a combined Dice and cross-entropy loss function, using the multi-class Dice formulation described by [14]). The Adam optimiser [41] was used with an initial learning rate of 3 × 10^− 4^ and batch size of two. Each epoch consisted of 250 training batches, and an exponential moving average of the training loss was computed during training. If this metric failed to improve by at least 5 × 10^− 3^ over 30 epochs, the learning rate was reduced by a factor of five. Each training fold was run for 1000 epochs.

All models were implemented in nnU-Net (v2.4.2) using Python (v3.9) and PyTorch (v2.3), supported by CUDA (v11.8). Training was performed on NVIDIA V100 or A40 GPUs with 250GB RAM and an Intel Xeon Gold 5320 (13 cores), using the M3 High-Performance Computing cluster [42].

### Model evaluation

All T1w models targeting WM- and BG-PVS were evaluated with stratified 5FCV procedures. The training dataset of 30 PVS segmentations was partitioned into five equal splits, with training performed on four splits and validated on the held-out split. Stratification ensured equal representation of images from each dataset in every fold, and data splits were kept consistent across all cross-validations (Sect. 2.4).

The T2w model was similarly trained and evaluated with a stratified 5FCV using 15 images from datasets C, HCP1200, and NIFD. The T1w+FLAIR model, trained on 10 images from dataset C, was evaluated with standard 5FCV, while the T2w+FLAIR model, trained on 10 images from datasets C and NIFD, was evaluated with a stratified 5FCV.

Finally, T1w models targeting MB-PVS (*n* = 60) and HP-PVS (*n* = 50) were also evaluated with a stratified 5FCV, conducted separately as each task used different datasets.

#### Evaluation metrics

The nnU-Net framework reports the Dice similarity coefficient (DSC) together with counts of true positives (TP), false positives (FP), and false negatives (FN). Additionally, we computed sensitivity (SEN) and positive predictive value (PPV), defined in Eq. (1). These metrics were derived from voxel-wise comparisons between manual reference segmentations and model predictions. DSC quantifies overall segmentation overlap. SEN measures the proportion of PVS voxels correctly identified. PPV measures the proportion of predicted voxels that were true PVS.

1 $$\begin{aligned} &\:DSC=\frac{2TP}{\left(TP\:+\:FP\right)\:+\:(TP\:+\:FN)},\\&\:SEN=\frac{TP}{TP\:+\:FN},\:PPV=\frac{TP}{TP\:+\:FP} \end{aligned}$$

To assess model performance at the cluster level, we used Lin’s concordance correlation coefficient (CCC), Bland-Altman analysis, and Spearman’s rank correlation. Lin’s CCC and Bland-Altman plots were used to assess agreement between predicted and actual values, whereas Spearman’s correlation measured monotonic relationships.

All quantitative metrics were computed for each label in their respective models: WM-PVS and BG-PVS in the T1w and T2w models; WM-PVS, BG-PVS, and WMH in the T1w+FLAIR and T2w+FLAIR models; and MB-PVS and HP-PVS in their dedicated models. Model predictions were manually reviewed against reference segmentations at multiple stages of development to evaluate segmentation accuracy and overall quality.

#### Statistical analysis

Normality of PVS metrics was evaluated with Shapiro-Wilk tests and inspection of Q-Q plots. All normally distributed numerical variables are presented as mean±standard deviation (SD), whereas non-normally distributed variables are presented as median (interquartile range, IQR). Lin’s CCC, Bland-Altman analyses, and non-parametric Spearman’s rank correlation tests were performed with the *epiR* (v2.0.74) package. Statistical significance level was set at *p* < 0.05. Statistical analyses and figure generation were performed using R (v4.3.3) and RStudio (v2023.12.1–402).

## Results

### T1w nnU-Net: WM and BG-PVS

#### Sparse annotation strategy

Initial model predictions produced segmentations with a median (IQR) PVS voxel count of 6,892.5 (3,697.5) and a cluster count of 432.5 (208.5). After manual correction, the segmentations contained a median of 6,812.5 (3,922.8) PVS voxels and 285.5 (140.5) clusters. In dataset C, 8 of 10 corresponding FLAIR scans showed WMHs confluent with PVS. Misclassification was also observed, with some cortical grey matter voxels incorrectly labelled as PVS, particularly evident in sagittal and coronal viewing planes.

#### Target spacing optimisation

**Fig. 7:**
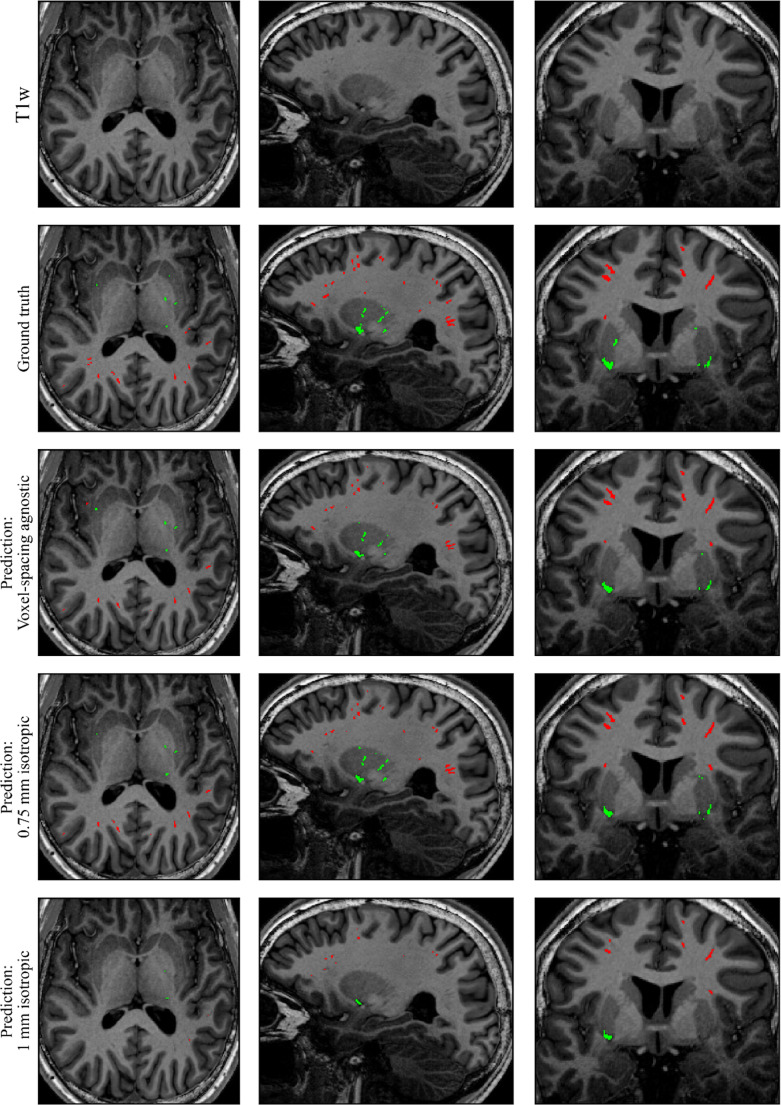
Comparison of model predictions to the manual PVS segmentation from Dataset A (7T, Siemens Magnetom, 0.75 mm isotropic voxel-spacing. Row 1: raw T1w image. Row 2: manual PVS segmentation overlaid on the T1w image. Row 3: prediction generated from the voxel-spacing agnostic nnU-Net. Row 4: prediction generated from the nnU-Net with 0.75 mm isotropic target spacing. Row 5: prediction generated form the nnU-Net with 1.0 mm isotropic target spacing. White matter PVS are labelled red. Basal ganglia PVS are labelled green

In general, performance was higher for models with higher resolution target spacing (smaller voxel size). Compared to alternative target spacings, the highest resolution model trained at 0.75 mm isotropic voxel-spacing performed the best (mean ± SD DSC = 55 ± 3%), followed by the 0.87 mm (DSC = 49.2 ± 1.9%), 0.80 mm (DSC = 46.2 ± 3.1%), and 1.0 mm (DSC = 40.5 ± 2.8%) isotropic voxel-spacing (Table 2). Notably, the *voxel-spacing agnostic* model substantially outperformed all models that considered voxel-spacing in image preprocessing (mean DSC = 64.3 ± 3.3%, DSC_WM−PVS_=70 ± 1.2%, and DSC_BG−PVS_=58.6 ± 5.7%). Visual inspection of PVS labels generated by the *voxel-spacing agnostic* model further reinforced the robust segmentation in both WM and basal ganglia regions (Fig. 7). Amongst the different models, the voxel-spacing agnostic model had the highest correlation and concordance metrics for both voxel counts (ρ = 0.94, Lin’s CCC = 0.79, 95%CI: 0.65–0.87) and cluster counts (ρ = 0.87, CCC = 0.86, 95%CI: 0.75–0.93).

**Table 2:** 5FCV DSC scores of T1w models targeting the WM and basal ganglia-PVS. Models in the spacing optimisation stage were evaluated with raw T1w MRI scans. In the preprocessing optimisation section, models were trained on images preprocessed with non-local means filtering (NLMF), adaptive histogram equalisation (AHE) or both NLMF and AHE. Segs_V2_ refers to the model trained and evaluated using segmentations derived from preprocessed T1w images (NLMF + AHE). The means and standard deviations represent performance metrics averaged across the cross-validation folds. Results are stratified by dataset across columns. Values are presented as Mean ± SD

Model	Overall	A	B	C
Spacing optimisation				
1.00 × 1.00 × 1.00 mm	40.5 ± 2.8	27.5 ± 2.1	33 ± 1.2	68.1 ± 4
0.87 × 0.87 × 0.87 mm	49.2 ± 1.9	35.8 ± 2.8	70.8 ± 2.5	48.3 ± 2.6
0.80 × 0.80 × 0.80 mm	46.2 ± 3.1	43.7 ± 3.7	48.6 ± 2.9	54.6 ± 4.4
0.75 × 0.75 × 0.75 mm	55.0 ± 3.0	67.3 ± 2.5	53.5 ± 3	57.4 ± 4.3
Agnostic	64.3 ± 3.3	66.7 ± 2.3	71.8 ± 2.9	69 ± 4.1
Preprocessing optimisation				
NLMF	63.6 ± 3.2	66.2 ± 2.5	71.3 ± 3.2	67.2 ± 4.9
AHE	63.4 ± 3.1	64.6 ± 2.2	71.4 ± 3	68.4 ± 4
NLMF + AHE	63.0 ± 3.1	65.2 ± 2.5	70.8 ± 3.2	67.3 ± 4.5
Segs_V2_	78.3 ± 1.7	78.4 ± 0.8	81 ± 2	80.2 ± 2.2

#### Image preprocessing optimisation

We evaluated the impact of image preprocessing on model performance. When trained on raw T1w MRI, the voxel-spacing agnostic nnU-Net achieved a mean DSC of 64.3 ± 3.3%. Preprocessing with NLMF (63.6 ± 3.2%), AHE (63.4 ± 3.1%) or their combination (63 ± 3.1%) did not improve performance (Table 2). Thus, the raw T1w model slightly outperformed all models with preprocessed image inputs. By contrast, training on segmentations derived from preprocessed T1w MRI (NLMF + AHE) images yielded substantially improved performance to 78.3 ± 1.7% DSC.

Performance was consistent across datasets and field strengths, with mean DSC of 78.4 ± 0.8% in 7 T (dataset A) and mean DSCs of 80.6 ± 2.1% in the 3 T datasets (dataset B: 81 ± 2%; dataset C: 80.2 ± 2.2%). Model predictions strongly agreed with manual annotations for both PVS voxel counts (ρ = 0.97, CCC = 0.98, 95%CI: 0.96–0.99) and cluster counts (ρ = 0.97, CCC = 0.9, 95%CI: 0.82–0.95). Bland-Altman plots (Supplementary Fig. 1–2) and correlation results (Supplementary Table 10) further supported these findings.

### T1w+FLAIR nnU-Net

**Fig. 8:**
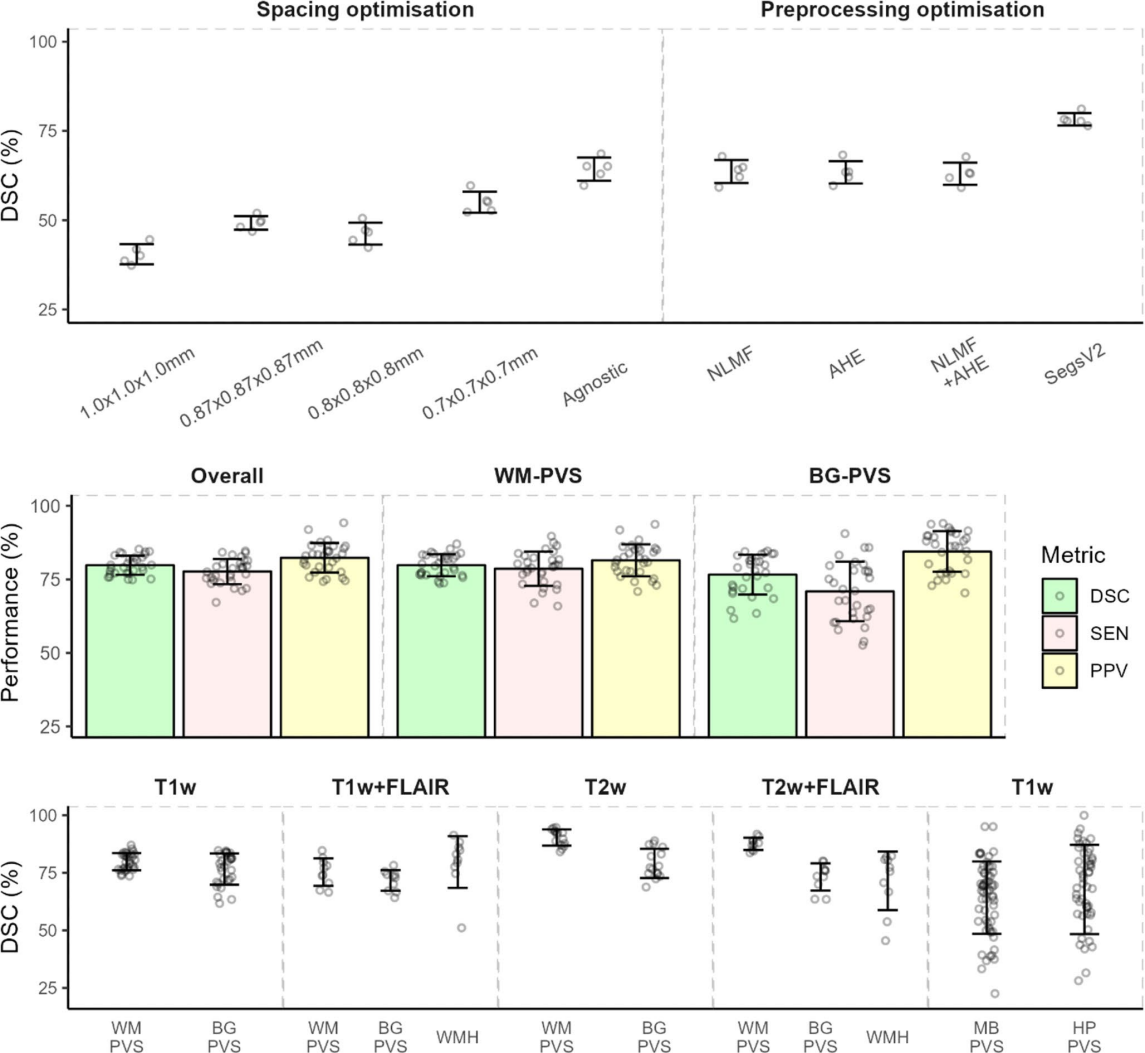
Performance of all nnU-Net models. Top-row: 5FCV mean ± SD Dice similarity coefficients for T1w nnU-Net models targeting the WM and BG-PVS. Middle-row: 5FCV metrics of the final T1w nnU-Net model. Performance is shown for the WM and BG-PVS (overall) (left), WM-PVS (middle), and BG-PVS (right). Bottom-row: region-wise DSC performance for the final T1w model (left), T1w+FLAIR model (middle-left), T2w model (middle), T2w+FLAIR model (middle-right), and T1w models for MB-PVS and HP-PVS (right)

In dataset C (*n* = 10, 5 females, age = 66.1 ± 8 years), the median (IQR) voxel counts were 1,590 (1,155) for WM-PVS, 523 (127) for BG-PVS, and 838.5 (488.5) for WMH. The overall mean DSC score was 75.6 ± 3.4%, with highest performance achieved for WMH (79.7 ± 7.6%), followed by WM-PVS (75.3 ± 4.4%), and BG-PVS (71.7 ± 3.1%) (Table 3; Fig. 8).

**Table 3:** Performance metrics for the final nnU-Net models evaluated via 5-fold cross validation. The means and standard deviations represent performance metrics averaged across the cross-validation folds. DSC=Dice-Similarity coefficient. SEN=Sensitivity. PPV=positive predictive value. Values are presented as Mean ± SD

Modality/Targets	DSC	SEN	PPV
T1w			
Overall	78.3 ± 1.7	77.9 ± 1.3	82.5 ± 2.6
WM-PVS	79.7 ± 0.7	79.1 ± 1.6	82.2 ± 2.9
BG-PVS	76.7 ± 3.1	70.5 ± 3.3	84.6 ± 2.3
T1w+FLAIR			
Overall	75.6 ± 3.4	70.3 ± 5.3	87.3 ± 3.6
WM-PVS	75.3 ± 4.4	69.4 ± 7.5	85.5 ± 3.5
BG-PVS	71.7 ± 3.1	61.8 ± 5.1	86.9 ± 4.9
WMH	79.7 ± 7.6	74.7 ± 6.2	89.6 ± 5.9
T2w			
Overall	84.7 ± 1.3	88.8 ± 2.1	92.7 ± 0.9
WM-PVS	90.4 ± 0.9	90.4 ± 1.8	93 ± 1.1
BG-PVS	79.1 ± 2	74.8 ± 8.1	88.7 ± 4.7
T2w+FLAIR			
Overall	77.5 ± 2.7	79.8 ± 6.3	88.4 ± 3.1
WM-PVS	87.6 ± 1.6	85.6 ± 2.7	88.8 ± 2
BG-PVS	73.2 ± 3.3	65.3 ± 4.3	84.4 ± 6
WMH	71.5 ± 6.1	67.3 ± 17.6	85.2 ± 15.7
T1w			
MB-PVS	64.3 ± 6.5	53.6 ± 8.6	81.1 ± 2.7
T1w			
HP-PVS	67.8 ± 5	57.6 ± 6.4	87.8 ± 5.9

### T2w nnU-Net

In T2w images (*n* = 15) across three datasets, the median voxel counts were 9,783 (14,634) for WM-PVS and 1,080 (1,048) for BG-PVS. The overall mean DSC was 84.7 ± 1.3%, with better performance for WM-PVS (90.4 ± 0.9%) compared to BG-PVS (79.1 ± 2%) (Table 3; Fig. 8).

### T2w+FLAIR nnU-Net

In the segmentations (*n* = 10), the median voxel counts were 7,931.5 (3,697.3) for WM-PVS, 704.5 (426.3) for BG-PVS, and 904 (2,550.5) for WMH. The overall mean 5FCV DSC was 77.5 ± 2.7%, with highest performance for WM-PVS (87.6 ± 1.6%), followed by BG-PVS (73.2 ± 3.3%), and WMH (71.5 ± 6.1%) (Table 3; Fig. 8).

### T1w nnU-Net: MB and HP-PVS

#### Midbrain-PVS segmentation

In the midbrain PVS segmentations (*n* = 60), the median PVS voxel and cluster counts were 53.5 (42.3) and 4 (2), respectively. The MB-PVS nnU-Net achieved a mean 5FCV DSC of 64.3 ± 6.5% (Table 3; Fig. 8). However, agreement with manual segmentations was poor for both voxel counts (ρ = 0.39, CCC = 0.19, 95%CI: 0.04–0.33) and cluster counts (ρ = 0.33, CCC = 0.29, 95%CI: 0.01–0.53).

#### Hippocampal-PVS segmentation

In the hippocampal PVS segmentations (*n* = 50), the median PVS voxel and cluster counts were 37.5 (39.5) and 4 (3), respectively. The HP-PVS nnU-Net achieved a mean 5FCV DSC of 67.8 ± 5% (Table 3; Fig. 8). Again, agreement with manual segmentations was poor for both voxel counts (ρ = 0.29, CCC = 0.11, 95%CI: −0.02–0.23) and cluster counts (ρ = 0.66, CCC = 0.63, 95%CI: 0.36–0.80). Bland-Altman plots (Supplementary Fig. 3) further illustrate these findings.

## Discussion

By combining the nnU-Net residual encoder with image-processing techniques, we developed a comprehensive pipeline for automated PVS segmentation in MRI. Using sparse annotations, we demonstrated that full-brain segmentations can be efficiently generated from partially labelled data. We further observed that voxel-spacing played an important role during image preparation, with resampling to non-native resolutions negatively impacting on performance. In contrast, a *voxel-spacing agnostic nnU-Net* yielded robust results across heterogeneous MRI datasets. Finally, we developed multimodal pipelines leveraging T1w, T2w, and FLAIR sequences for PVS segmentation including pilot models for PVS segmentation in the midbrain and hippocampus. Together, these models constitute a versatile toolkit for comprehensive assessment of perivascular spaces in MRI.

### T1w nnU-Net: WM + BG-PVS

#### Sparse annotation strategy

A major challenge in medical image segmentation tasks is the acquisition of high-quality labels, as manual annotation is highly time-consuming. Previous MRI-PVS segmentation models have typically used small training datasets (≤ 40 images) [11], or were validated on synthetic MRI scans with simulated PVS structures [43–45]. We addressed this challenge using a sparse annotation strategy to efficiently generate PVS segmentations.

Sparse annotation strategies are a recent innovation for efficiently acquiring labelled training data. Gotkowski et al., (2024) adapted the nnU-Net to complete multi-organ segmentations from manually drawn scribbles outlining abdominal organs in CT scans [25]. Here, we present the first application of this approach to MRI-PVS, demonstrating that sparse annotation can produce high-quality segmentation. This strategy offers a scalable solution for future studies requiring large numbers of PVS segmentations.

During manual annotation, the choice of annotation plane was found to influence the resulting segmentations. Although annotations were initially performed in the axial plane, this approach had notable limitations. Some structures that appeared to be PVS in the axial view were revealed to be cortical grey matter when examined in the coronal or sagittal planes. Additionally, certain PVS, particularly in the superior brain regions, appeared as small, round foci on axial slices but displayed linear or elongated morphologies in the coronal and sagittal views, where they were substantially easier to identify and annotate accurately. These observations highlight the importance of using multiple annotation planes to confirm the nature of each lesion or selecting the viewing plane that best captures the morphology of the structure of interest.

#### Target voxel-spacing optimisation

Target voxel-spacing is a critical factor in MRI-PVS segmentation. Most previous models were trained on a single homogenous image dataset and resampled scans to a fixed resolution (e.g., 1.0 mm isotropic) [46]. By default, the nnU-Net selects the median voxel-spacing of the training set. However, such resampling introduces artefacts and degrades segmentation performance.

Furthermore, to identify the most robust and generalisable resolution, we benchmarked nnU-Net models across multiple target voxel-spacings (0.75, 0.8, 0.87, 1.0 mm isotropic) and an agnostic variant. The voxel-spacing agnostic model achieved the most generalisable performance (Fig. 8), avoiding the resampling artefacts caused by fixed-resolution approaches. This strategy enhances model robustness across heterogeneous MRI acquisitions, including scans obtained at different field strengths, and offers a practical framework for future automated PVS segmentation studies.

A recent study has evaluated the use of nnU-Net for PVS segmentation in T1w MRI acquired from multiple scanners (mcPVS-Net) [47]. Similar to Huang et al. (2024) our models are based on the nnU-Net framework and achieved comparable overall DSC performance (~ 80%) in T1w MRI for WM- and BG-PVS. However, their training dataset was restricted to a homogenous acquisition protocol (3T, 1 mm isotropic resolution), which likely limits generalisability to higher-resolution MRI. Importantly, by incorporating higher-resolution images into training, our approach extends prior work and supports broader generalisability and more reliable applications across diverse MRI parameters and acquisition protocols.

#### Image preprocessing

Several preprocessing techniques have been used to enhance PVS visibility in MRI. Gamma contrast adjustment improves visual distinction between PVS and background, facilitating manual grading [48]. Non-local means filtering denoises images and can reduce the number of false positives from the Frangi filter [49]. Contrast-limited adaptive histogram equalisation has likewise been applied to T2w MRI to enhance PVS visibility and support segmentation with a support vector machine classifier [50].

Similarly, we integrated image enhancement techniques to facilitate both automated detection and manual annotation of MRI-visible PVS. Specifically, non-local means filtering was applied to reduce background noise, followed by adaptive histogram equalisation to enhance PVS contrast relative to surrounding structures. The preprocessing produced clearer boundaries between PVS and adjacent white matter (Fig. 3). Although models trained on preprocessed T1w images did not outperform the model trained on raw images (Table 2), training the nnU-Net on segmentations derived from preprocessed images resulted in a substantial performance gain (+ 14% DSC).

The result suggests that manual segmentations derived from preprocessed images yield more consistent, higher-quality labels, leading to more reliable models. This benefit likely reflects the sharper boundaries between PVS and the surrounding background (Fig. 3). When combined with sparse annotation strategies, this approach provides an efficient method of generating high-quality labels for PVS segmentation, with potential applicability to a broad range of medical image segmentation tasks.

Conversely, the preprocessing techniques may have distorted the boundaries between PVS and the surrounding tissue, potentially reducing segmentation quality and model performance. However, this drawback appeared to be offset by the increased consistency of the PVS labels, as reflected in the improved performance metrics.

### T2w nnU-Net

PVSs are more prominent and readily visualised on T2w than on T1w MRI. Although T1w scans are more widely available, most prior segmentation studies have focussed on the T2w sequence [7, 9, 51–53]. A key contribution of our work is the development of a model trained across three diverse datasets, incorporating a heterogeneous sample of healthy individuals and patients.

Consistent with the superior visibility of PVS on T2w MRI, models trained from T2w segmentations achieved higher DSC performance (+ 5%) compared to T1w models. The improvement, however, varied by region: WM-PVS showed nearly a 10% increase in DSC with T2w models, whereas BG-PVS demonstrated only a marginal gain (+ 1%) (Table 3). Although PVS visibility is enhanced on T2w MRI, improvements in BG-PVS segmentation were likely constrained by the relatively small number of BG-PVS examples available for model training. In contrast, performance gains for WM-PVS may have been facilitated by their higher prevalence. Importantly, these findings should be interpreted with caution given the small datasets used in the 5-fold cross-validation, which limits statistical robustness and reduces the reliability of performance comparisons between the T1w and T2w models.

### Multimodal nnU-Nets

Rashid et al. (2023) developed a multimodal convolutional neural network using T1w and FLAIR for PVS segmentation [54]. Beyond architectural differences, our approach offers two key advantages. First, we introduced region-specific labels for WM- and BG-PVS, enabling quantification of regional metrics without the need for additional tissue parcellation tools such as FreeSurfer. Second, our dual-channel T1w+FLAIR nnU-Net simultaneously segments WMHs and PVS, eliminating the need for supplementary WMH detection modules (e.g., Lesion Segmentation Tool) to address lesion-related confounds. This integrated framework generates segmentation maps that facilitate direct assessment of the topological relationships between PVS and WMH, two key markers of cerebral small vessel disease.

Furthermore, our T2w+FLAIR model combines the advantages of both sequences: T2w scans, which provide optimal visibility of PVS compared to T1w, and FLAIR scans, which facilitate the detection of WMHs. The T2w+FLAIR model was trained on MRI data from both controls and patients with frontotemporal dementia, increasing its potential generalisability relative to our T1w+FLAIR model. By integrating multimodal inputs, these models provide complementary information that may help disentangle PVS from WMH lesions. This could support future studies aimed at more accurately distinguishing and quantifying these lesions when they co-occur, and at probing whether they share overlapping mechanisms of progression.

### T1w nnU-Net: MB and HP-PVS

To date, studies of midbrain and hippocampal PVS have relied on coarse measures such as cluster counts or binarised presence/absence scores [36, 55]. Midbrain PVS enlargement has been reported in Parkinson’s disease compared with age- and sex- matched controls [56], while hippocampal PVS enlargement has been linked to ageing and hypertension [57, 58]. However, associations between hippocampal PVS and cognition, mild cognitive impairment, or vessel pulsatility index have been inconsistent, with several studies reporting no significant relationships [59, 60]. These inconsistencies potentially reflect the coarse nature of prior PVS measures. Our novel nnU-Net models provide voxel-wise quantification of PVS in the midbrain and hippocampus, enabling more detailed investigations of their role in health and disease.

### Limitations

One limitation of this study is the relatively small sample size used for validation. Robust machine learning requires datasets of sufficient size and diversity [23]. Our T1w model for WM- and BG-PVS was validated with only 30 segmentations. Despite this, the model demonstrated robust accuracy across heterogenous MRI datasets.

A limitation of our models is the small datasets used for their training and evaluation. Deep learning approaches typically require large and diverse datasets to achieve robust and generalisable performance. Therefore, these models should be considered proof-of-concept implementations rather than fully validated tools. As such, they serve as an initial foundation for MRI-based PVS segmentation and quantification. Future work is required to establish generalisability, including training on larger cohorts, validation on independent external datasets, and systematic benchmarking against existing methods.

Another limitation is that the validation dataset consisted of healthy controls rather than patients with neurological disorders. Inclusion of disease populations in future studies will be important to ensure the model can accurately segment PVS in the presence of pathological lesions. Furthermore, rigorous evaluation on independent datasets acquired at image resolutions not represented in the training data is required to robustly establish the model’s generalisability. While the current results demonstrate performance within the available datasets, the reliability of PVS detection in scans obtained at different spatial resolutions or with differing acquisition parameters remains to be empirically determined.

A key limitation of the T1w+FLAIR model is that it was trained on a single dataset with 1 mm isotropic resolution. Thus, its generalisability to external datasets and different MRI acquisition protocols remains unvalidated. To improve model generalisability, future work should expand the training to more heterogeneous datasets, including both higher resolution scans and images from patient populations. Similar extensions would also benefit the T2w+FLAIR model. Additionally, semi-supervised learning approaches, as demonstrated in Sect. 3.1.4, could offer a cost-effective strategy to increase dataset size and diversity while improving model performance.

Our pilot models for midbrain and hippocampal PVS segmentation could be improved in several ways. First, additional labelled training data are needed, as the dataset used here (*n* = 50–60) is small by biomedical image segmentation standards [23]. Second, preprocessing techniques such as NLMF and AHE should be explored as potential strategies to improve PVS segmentation in these regions.

## Conclusions

Enlarged perivascular spaces are emerging as an important imaging biomarker for neurological diseases. In this study, we introduced sparse annotation as an efficient strategy for generating PVS segmentations in the white matter and basal ganglia of both T1w and T2w MRI. Preprocessing further enhanced annotation quality and consistency, yielding improved model performance when used for training. We also optimised multimodal models that integrate FLAIR with either T1w or T2w MRI, enabling simultaneous detection of PVS and white matter hyperintensities. Additionally, we developed pilot nnU-Net models for automated PVS segmentation in the midbrain and hippocampal regions of T1w MRI. By releasing these models openly, we provide the research community with a reproducible framework for perivascular space investigations in MRI, which can be further fine-tuned and adapted for new datasets.

## Supplementary information

Below is the link to the electronic supplementary material.

Supplementary File 1 (DOCX 1.64 MB)

Supplementary File 2 (XLSX 24.8 KB)

## Data Availability

Our nnU-Net models are open-source and available at: [https://github.com/wpham17/nnUNet-Perivascular-Spaces](https://github.com/wpham17/nnUNet-Perivascular-Spaces).
