# Supplementary material for: A comprehensive framework for automated segmentation of perivascular spaces in brain MRI with the nnU-Net
Source: Neuroradiology. 2026 Apr 17;68(6):1465–83. doi: 10.1007/s00234-026-03993-y (PMC13323141; doi:10.1007/s00234-026-03993-y)
Supplement: Supplementary file 1 — (DOCX 1.64 MB) [file 234_2026_3993_MOESM1_ESM.docx]

# Supplementary Methods Section 1.

The open-access datasets included the Alzheimer's Disease Neuroimaging Initiative (ADNI3), Australian Imaging Biomarkers and Lifestyle (AIBL) study, Human Connectome Project (HCP1200), frontotemporal lobar degeneration neuroimaging initiative (FTLDNI/NIFD), and Open Access Series of Imaging Studies (OASIS-3) (Ellis et al., 2009; Glasser et al., 2013; Jack et al., 2008; LaMontagne et al., 2019; Van Essen et al., 2012, 2013).

Data used in the preparation of this article were obtained in part from the Alzheimer's Disease Neuroimaging Initiative (ADNI) database (adni.loni.usc.edu) and the Aging Brain: Vasculature, Ischemia, and Behavior Study database (ABVIB). The ADNI was launched in 2003 as a public-private partnership, led by Principal Investigator Michael W. Weiner, MD. The ABVIB study was launched in 1994 as a NIA-funded program project led by Principal Investigator Helena C. Chui, MD. Data from AIBL was collected by the AIBL study group. AIBL study methodology has been reported previously (Ellis et al., 2009). NIFD/FTLDNI was funded through the National Institute of Aging and started in 2010. The Principal Investigator of NIFD was Dr. Howard Rosen, MD at the University of California, San Francisco. The data are the result of collaborative efforts at three sites in North America. For up-to-date information on participation and protocol, please visit <http://memory.ucsf.edu/research/studies/nifd>.

# Supplementary Methods Section 2.

To complement the voxel-wise evaluation metrics, we assessed cluster-level performance using the Dice similarity coefficient, sensitivity, and positive predictive value (Eq. 1). These metrics were computed for the final model from each major section. Cluster-level evaluation was performed using overlap detection thresholds of 1, 2, 3, 4, and ≥ 5 voxels.

For a given reference cluster, the cluster was considered a true positive (TP) if the predicted segmentation overlapped with at least *k* voxels of that reference cluster, where *k* corresponded to the detection threshold. If the overlap was < *k* voxels, the reference cluster was considered a false negative. Predicted clusters that did not overlap with any reference cluster were classified as false positives (FP). At each evaluated threshold, clusters smaller than *k* voxels were excluded from analysis. The results are presented in Supplementary Tables 11-13.

**Supplementary Table 1.** Scanning parameters for T1-weighted (T1w) MRI sequences used for model development and perivascular space segmentation. Ten images per dataset were selected for manual segmentation of PVS in the white matter and basal ganglia. These segmentations comprised the training dataset for development of the T1w nnU-Net (Section 2.4). MPRAGE=magnetisation-prepared rapid gradient sequence; TR=Repetition Time; TE=Echo Time; TI=Inversion Time; FA=Flip Angle.

|  | **Dataset A** | **Dataset B** | **Dataset C** | |
| --- | --- | --- | --- | --- |
| Modality | T1w | T1w | T1w | FLAIR |
| Sequence | MPRAGE | MPRAGE | MPRAGE |  |
| Field Strength | 7T | 3T | 3T | 3T |
| Manufacturer | Siemens | Philips | Siemens | Siemens |
| Model | Magnetom | Achieva | Trio Tim | Trio Tim |
| Number of Head Coils | 32 | 21 | 12 | 12 |
| Acquisition Plane | Sagittal | Axial | Sagittal | Sagittal |
| Field of View (mm) | 240.0 × 167.25 | 250.56 × 174.0 | 256 × 256 | 512 × 512 |
| Voxel spacing (mm) | 0.75 × 0.75 × 0.75 | 0.87 × 0.87 × 0.87 | 1.0 × 1.0 × 1.0 | 1.0 × 1.0 × 1.0 |
| Slice Thickness (mm) | 0.75 | 0.87 | 1.0 | 1.0 |
| Voxel size (mm^3^) | 0.422 | 0.659 | 1.0 | 1.0 |
| TR (ms) | 5000 | 5600 | 1900 | 6000 |
| TE (ms) | 3.1 | 2.5 | 2.55 | 380 |
| TI (ms) | - | - | 900 | 2100 |
| FA (°) | 4 | 8 | 9 | 120 |

**Supplementary Figure 1.** Bland-Altman plots evaluating agreement between reference and predicted PVS voxel counts for all T1w models targetting white matter (WM) and basal ganglia (BG) PVS. Differences are plotted against the mean of the reference and predicted values. Row 1: Models in the voxel spacing optimisation stage. From left to right: target voxel spacings of 1.00×1.00×1.00 mm, 0.87×0.87×0.87 mm, 0.8×0.8×0.8 mm, 0.75×0.75×0.75 mm, and a voxel spacing agnostic model (blue). Row 2: Models in the image preprocessing optimisation stage. From left to right: the model trained with raw images, non-local means filtering (NLMF), adaptive histogram equalisation (AHE), and combined NLMF+AHE preprocessing (blue). Row 3: Model trained and evaluated with revised manual segmentations using NLMF+AHE (blue).


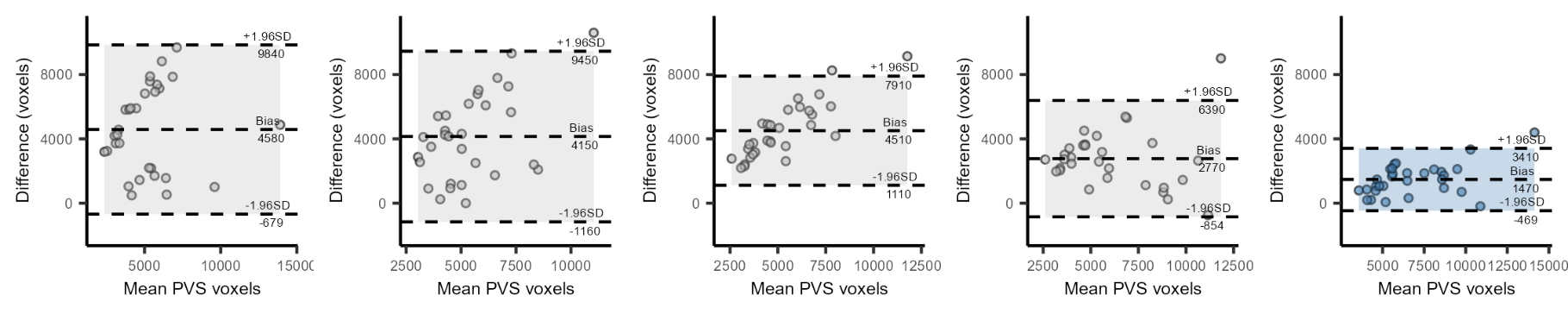

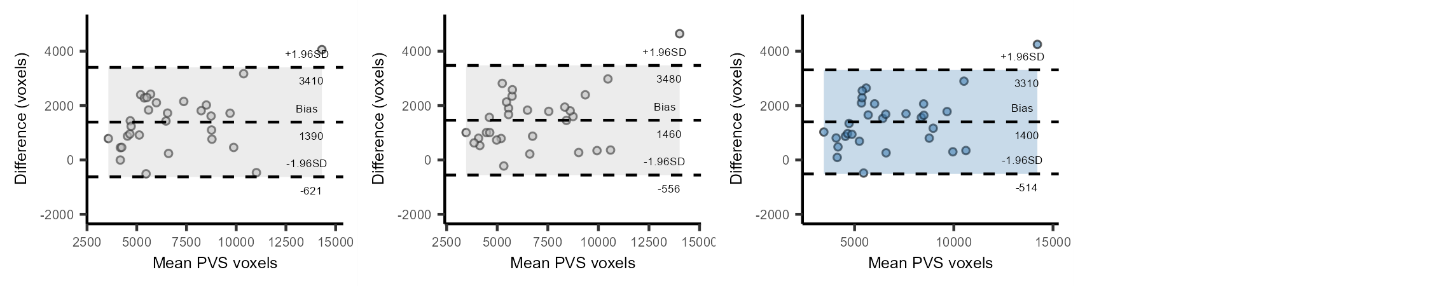

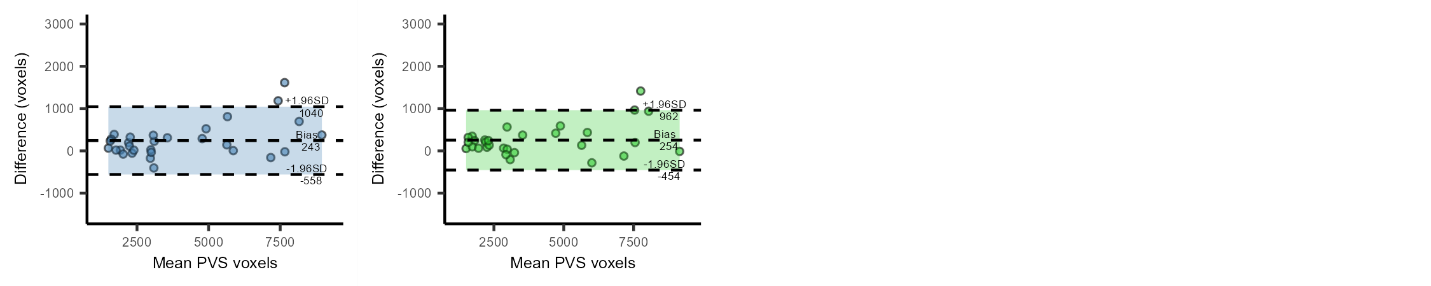


**Supplementary Figure 2.** Bland-Altman plots evaluating agreement between reference and predicted PVS cluster counts for all T1w models targetting white matter (WM) and basal ganglia (BG) PVS. Differences are plotted against the mean of the reference and predicted values. Row 1: Models in the voxel spacing optimisation stage. From left to right: target voxel spacings of 1.00×1.00×1.00 mm, 0.87×0.87×0.87 mm, 0.8×0.8×0.8 mm, 0.75×0.75×0.75 mm, and a voxel spacing agnostic model (blue). Row 2: Models in the image preprocessing optimisation stage. From left to right: the model trained with raw images, non-local means filtering (NLMF), adaptive histogram equalisation (AHE), and combined NLMF+AHE preprocessing (blue). Row 3: Model trained and evaluated with revised manual segmentations using NLMF+AHE (blue).


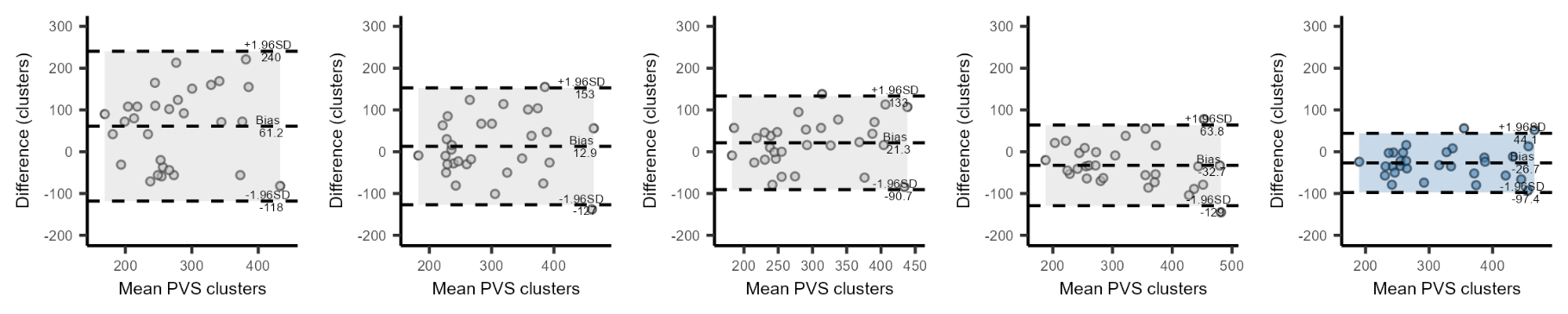

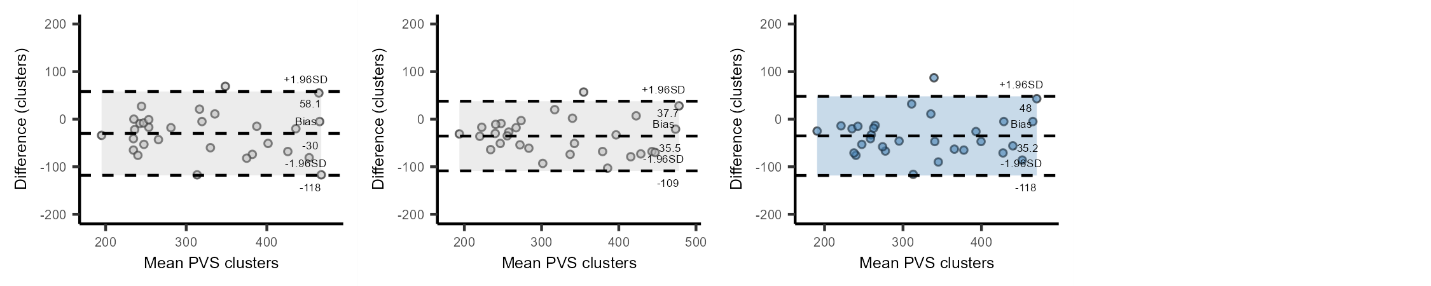

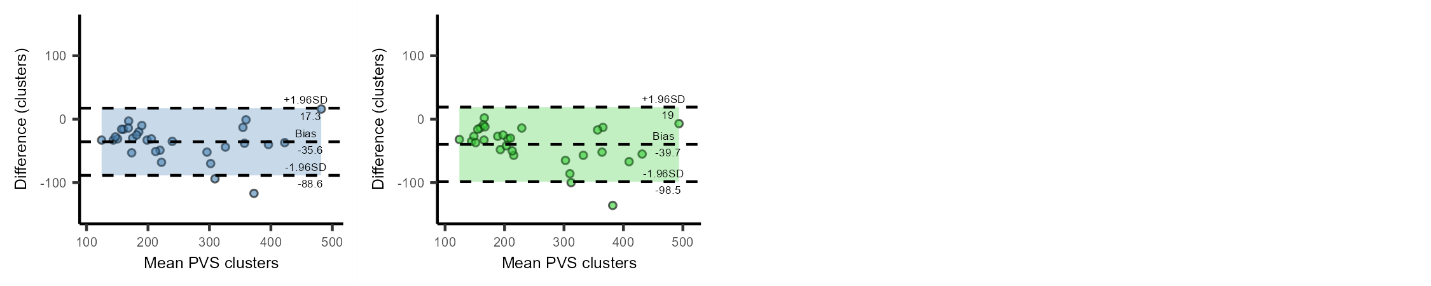


**Supplementary Figure 3.** Bland-Altman plots evaluating agreement between automated T1w models targetting PVS segmentaton in the midbrain and hippocampus. Differeneces in total PVS voxel counts (left panels) and cluster counts (right panels) are plotted against their means. Solid lines indicate the mean bias, and dashed lines represent the 95% confidence intervals.


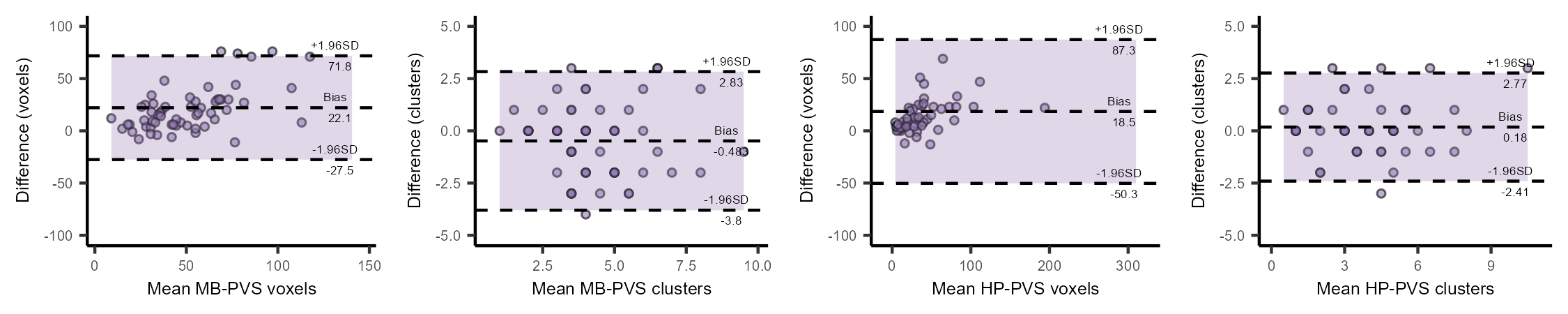


# Acknowledgements

**ADNI**

Data collection and sharing for the Alzheimer's Disease Neuroimaging Initiative (ADNI) is funded by the National Institute on Aging (National Institutes of Health Grant U19AG024904). The grantee organization is the Northern California Institute for Research and Education. In the past, ADNI has also received funding from the National Institute of Biomedical Imaging and Bioengineering, the Canadian Institutes of Health Research, and private sector contributions through the Foundation for the National Institutes of Health (FNIH) including generous contributions from the following: AbbVie, Alzheimer's Association; Alzheimer's Drug Discovery Foundation; Araclon Biotech; BioClinica, Inc.; Biogen; Bristol-Myers Squibb Company; CereSpir, Inc.; Cogstate; Eisai Inc.; Elan Pharmaceuticals, Inc.; Eli Lilly and Company; EuroImmun; F. Hoffmann-La Roche Ltd and its affiliated company Genentech, Inc.; Fujirebio; GE Healthcare; IXICO Ltd.; Janssen Alzheimer Immunotherapy Research & Development, LLC.; Johnson & Johnson Pharmaceutical Research & Development LLC.; Lumosity; Lundbeck; Merck & Co., Inc.; Meso Scale Diagnostics, LLC.; NeuroRx Research; Neurotrack Technologies; Novartis Pharmaceuticals Corporation; Pfizer Inc.; Piramal Imaging; Servier; Takeda Pharmaceutical Company; and Transition Therapeutics.

**AIBL**

Data used in the preparation of this article was obtained from the Australian Imaging Biomarkers and Lifestyle flagship study of ageing (AIBL) funded by the Commonwealth Scientific and Industrial Research Organisation (CSIRO) which was made available at the ADNI database (www.loni.usc.edu/ADNI). The AIBL researchers contributed data but did not participate in analysis or writing of this report. AIBL researchers are listed at [www.aibl.csiro.au](http://www.aibl.csiro.au).

**HCP1200**

Data were provided in part by the Human Connectome Project, WU-Minn Consortium (Principal Investigators: David Van Essen and Kamil Ugurbil; 1U54MH091657) funded by the 16 NIH Institutes and Centers that support the NIH Blueprint for Neuroscience Research; and by the McDonnell Center for Systems Neuroscience at Washington University.

**NIFD**

Data collection and sharing for the NIFD project was funded by the Frontotemporal Lobar Degeneration Neuroimaging Initiative (National Institutes of Health Grant R01 AG032306). The study is coordinated through the University of California, San Francisco, Memory and Aging Center. FTLDNI data are disseminated by the Laboratory for Neuro Imaging at the University of Southern California.

**OASIS-3**

Data were provided in part by OASIS-3: Longitudinal Multimodal Neuroimaging: Principal Investigators: T. Benzinger, D. Marcus, J. Morris; NIH P30 AG066444, P50 AG00561, P30 NS09857781, P01 AG026276, P01 AG003991, R01 AG043434, UL1 TR000448, R01 EB009352. AV-45 doses were provided by Avid Radiopharmaceuticals, a wholly owned subsidiary of Eli Lilly.

# References

Ellis, K. A., Bush, A. I., Darby, D., De Fazio, D., Foster, J., Hudson, P., Lautenschlager, N. T., Lenzo, N., Martins, R. N., Maruff, P., Masters, C., Milner, A., Pike, K., Rowe, C., Savage, G., Szoeke, C., Taddei, K., Villemagne, V., Woodward, M., … Yastrubetskaya, O. (2009). The Australian Imaging, Biomarkers and Lifestyle (AIBL) study of aging: Methodology and baseline characteristics of 1112 individuals recruited for a longitudinal study of Alzheimer’s disease. *International Psychogeriatrics*, *21*(4), 672–687. https://doi.org/10.1017/S1041610209009405

Glasser, M. F., Sotiropoulos, S. N., Wilson, J. A., Coalson, T. S., Fischl, B., Andersson, J. L., Xu, J., Jbabdi, S., Webster, M., Polimeni, J. R., Van Essen, D. C., & Jenkinson, M. (2013). The minimal preprocessing pipelines for the Human Connectome Project. *NeuroImage*, *80*, 105–124. https://doi.org/10.1016/j.neuroimage.2013.04.127

Jack, C. R., Bernstein, M. A., Fox, N. C., Thompson, P., Alexander, G., Harvey, D., Borowski, B., Britson, P. J., Whitwell, J. L., Ward, C., Dale, A. M., Felmlee, J. P., Gunter, J. L., Hill, D. L. G., Killiany, R., Schuff, N., Fox-Bosetti, S., Lin, C., Studholme, C., … Weiner, M. W. (2008). The Alzheimer’s Disease Neuroimaging Initiative (ADNI): MRI methods. *Journal of Magnetic Resonance Imaging*, *27*(4), 685–691. https://doi.org/10.1002/jmri.21049

LaMontagne, P. J., Benzinger, T. LS., Morris, J. C., Keefe, S., Hornbeck, R., Xiong, C., Grant, E., Hassenstab, J., Moulder, K., Vlassenko, A. G., Raichle, M. E., Cruchaga, C., & Marcus, D. (2019). OASIS-3: Longitudinal Neuroimaging, Clinical, and Cognitive Dataset for Normal Aging and Alzheimer Disease. *medRxiv*, 2019.12.13.19014902. https://doi.org/10.1101/2019.12.13.19014902

Van Essen, D. C., Smith, S. M., Barch, D. M., Behrens, T. E. J., Yacoub, E., & Ugurbil, K. (2013). The WU-Minn Human Connectome Project: An overview. *NeuroImage*, *80*, 62–79. https://doi.org/10.1016/j.neuroimage.2013.05.041

Van Essen, D. C., Ugurbil, K., Auerbach, E., Barch, D., Behrens, T. E. J., Bucholz, R., Chang, A., Chen, L., Corbetta, M., Curtiss, S. W., Della Penna, S., Feinberg, D., Glasser, M. F., Harel, N., Heath, A. C., Larson-Prior, L., Marcus, D., Michalareas, G., Moeller, S., … Yacoub, E. (2012). The Human Connectome Project: A data acquisition perspective. *NeuroImage*, *62*(4), 2222–2231. https://doi.org/10.1016/j.neuroimage.2012.02.018
